# Prevalence and patterns of tobacco and/or nicotine product use in Japan (2017) after the launch of a heated tobacco product ( *IQOS* ^®^): a cross-sectional study

**DOI:** 10.12688/f1000research.52407.2

**Published:** 2022-03-21

**Authors:** Esther F. Afolalu, Peter Langer, Karina Fischer, Steve Roulet, Pierpaolo Magnani

**Affiliations:** 1 PMI R&D, Philip Morris Products S.A, Neuchâtel, Switzerland; 2 Philip Morris Products S.A., Lausanne, Switzerland

**Keywords:** Japan, smoking, heat-not-burn, heated tobacco product, IQOS, cross-sectional survey

## Abstract

**Background: **Several smoke-free tobacco and/or nicotine-containing products (TNP) have emerged in recent years to support tobacco harm reduction strategies and reduce individual health risks and population harm relative to continued cigarette smoking. This paper describes the nationwide prevalence and patterns of TNP use in Japan following the commercialization of one such smoke-free TNP, the heated tobacco product *IQOS*® (Philip Morris International).

**Methods:** We analyzed the first annual data (2016–2017) of two repeated cross-sectional surveys conducted in a representative sample of the Japanese general adult (≥20 years of age) population (N = 4,878) and a sample of adult *IQOS* users (N = 2,000). We assessed the prevalence of current TNP use according to type of product (cigarettes, *IQOS*, e-cigarettes, and other TNPs) in the general population and patterns of TNP use in the *IQOS* user sample.

**Results: **The prevalence of current use across all TNP in the sampled general population was 18.5% (95% confidence interval 17.2–19.5%), with 17.5% (16.4–18.6%) for cigarette smoking and 1.8% (1.4–2.2%) for *IQOS* use. Regarding the distribution of patterns of use in the *IQOS* user survey, the majority (63.4% [61.2–65.6%]) were exclusive users of *IQOS*, followed by 20.6% (18.7–22.5%) of individuals who reported dual use of *IQOS* and cigarettes.

**Conclusions:** In Japan, cigarette smoking remains the most prevalent way of consuming TNP; however, *IQOS* is being adopted by a growing number of adult Japanese smokers. These findings serve as baseline data for monitoring trends over time in the use and adoption of potential smoke-free TNP in Japan.

## Introduction

Tobacco harm reduction aims to complement tobacco control efforts and reduce population harm by switching smokers to less-harmful smoke-free tobacco and/or nicotine-containing products (TNP). A key part of tobacco harm reduction is also to prevent initiation or re-initiation of TNP use by never and former users, respectively. ^1– 3^ Unlike cigarettes, which burn tobacco and produce a complex mixture of harmful and potentially harmful constituents (HPHC) as a result of combustion, *IQOS* ^®^—a smoke-free TNP developed by Philip Morris International (PMI) ^4^—heats tobacco to temperatures below the level of combustion, producing an aerosol with markedly reduced levels of HPHCs. The aerosol generated from *IQOS* has, on average, 90–95% lower levels of HPHCs than cigarette smoke but provides comparable nicotine uptake and delivery. ^5^ Consequently, smokers switching completely to *IQOS* are exposed to much lower levels of HPHCs than those who continue smoking cigarettes. ^6, 7^

Since its launch in 2014 in Japan, *IQOS* has become available in 63 markets around the world, and approximately 14 million adult smokers have already switched to *IQOS* use. ^4^ The expansion in *IQOS* availability raises the need for monitoring its use by the population with the aim of informing public health advocates about its prevalence and use patterns, which will further enable regulators to ascertain its role in harm reduction as a substitute for cigarettes. ^8, 9^ Only a few national surveillance surveys, including the DEBRA study in Germany ^10^ and the Japan National Health and Nutrition Survey, ^11^ have been updated to report the prevalence of use of heated tobacco products, such as *IQOS,* and the adoption of these products by smokers.

Tabuchi *et al.* pioneered an attempt to describe the prevalence of smoke-free TNP use in Japan by using the *Japan “Society and New Tobacco” Internet Survey* (JASTIS), ^12^ a longitudinal study of 8,240 individuals from 2015 to 2017. At baseline, 48% of JASTIS participants reported awareness of existence of these products, and 1.3% reported current use (use in the last 30 days) of e-cigarettes and heated tobacco products, with the current use of *IQOS* increasing from 0.3% in 2015 to 3.6% in 2017. ^13, 14^ A recent pilot cross-sectional survey of 4,154 TNP consumers in Tokyo, Osaka, and Sendai also shed light on TNP usage following the introduction of heated tobacco products in Japan. ^15^ In contrast to the JASTIS survey, this study defined current use as ever use and consumption of at least 100 cigarettes or equivalent of other TNP rather than use in the last 30 days. The authors reported usage of heated tobacco products (IQOS, glo, and Ploom) by 5% of the participants, 67% of whom were IQOS users. In the sample of current TNP users in the survey, 16% reported exclusive use of heated tobacco products and 11% reported dual use with cigarettes. While these studies provided some insight into the use of *IQOS* and other TNP, they were not based on a representative sample of the Japanese general adult population or they were focused on specific regions, which limited the possibility of obtaining an accurate and unbiased estimation of the nationwide prevalence of TNP use in Japan. ^16– 18^

This paper, therefore, reports the results of a population-based survey (2016-2017) on TNP use in Japan since the launch of *IQOS.* The main goal of this survey was to determine the nationwide prevalence of *IQOS* use in Japan following its introduction in the Japanese market. A second survey of individuals who were purchasers of *IQOS* was also conducted with the aim of describing the use patterns of *IQOS*, alone or in combination with other TNP. From a tobacco harm reduction perspective, in addition to assessing prevalence, it is also important to understand whether smoke-free TNP are being used alone or in combination with other TNP, especially combustible TNP such as cigarettes, and if those who had never used any TNP are now using these products to initiate TNP use. Considering that *IQOS* was fairly new in the Japanese TNP marketplace when the study was initiated, its prevalence of use was expected to be low, as illustrated by previous surveys; ^13, 15^ likewise, the sample size of *IQOS* users to be recruited for the survey was also expected to be low. Obtaining a sufficient sample size for reliable estimation of the combined use of products and use patterns would have been prohibitively expensive in a nationwide population-based survey. Moreover, surveying the general adult population alone might only reflect a partial story of use at a pivotal time marking early adoption of the product. This might not always be the most practical means of gathering detailed information on product use and use patterns displayed by legal age (≥20 years of age) users who started using the product soon after it was available and before the majority of other smokers in the general population switched (i.e., “early adopters”). ^19^

## Methods

### Study design and setting

Cross-sectional surveys in both general population and *IQOS* user sample were initiated in a representative sample of the Japanese general adult population and in a sample of adult *IQOS* users in Japan in December 2016. Since then, these surveys were repeated annually over a period of three years. Each annual sampling of the surveys consisted of four approximately equal-sized waves of participants spaced throughout the year(s) to account for potential seasonal differences. The current paper analyzed the data from the first year of the surveys, which comprised four waves of fieldwork (i.e., Wave 1 in December 2016 and Waves 2, 3, and 4 in March, May, and July 2017, respectively). The fieldwork was coordinated and run by third-party suppliers (Ipsos Mori UK Ltd./Ipsos Japan).

### Participants

*Sampling: general population survey*

The general adult population sample was obtained by means of a multi-purpose survey (Omnibus). The Omnibus is a syndicated survey with multiple participating organizations and companies and is divided into different sections; therefore, the topics covered might change from wave to wave. The Omnibus was based on a three-stage stratified proportional sampling strategy that included the whole country. At the first stage, sampling points (from the census units) were allocated among the 12 Japanese administrative regions on the basis of their share of the population. A total of 157 sampling points were selected for an initial sample selection of 4,000 addresses. The second stage involved the use of an electronic residential map for identifying households within each sampling point, followed by random selection of about 40 households within the sampling points. The third stage sampled respondents within the selected households. Respondents were visited and approached by interviewers who confirmed and recorded consent of the individuals to take part in the study. Participants had to meet the inclusion criteria of the study: be legally authorized to buy tobacco products in Japan (i.e., ≥20 years of age); be current residents of Japan; be able to read, write, and understand Japanese; and be willing to participate in the study. Within each sampling point, broad quotas on age and sex were set to ensure representativeness of the Japanese population (e.g. a minimum target of having at least 1 male aged 20-29 years from a sampling point).

For the purpose of the present study, the “Tobacco Use Prevalence” paper questionnaire was developed for this study similarly to several existing tobacco and/or nicotine product use questions in the literature ^12^ to capture information about product use and was a stand-alone section handed to the participants for self-completion. The interviewer was available in case of queries and to check the section for completeness. The mode of administration for other questionnaires in the Omnibus is generally through face-to-face interview with a paper questionnaire that is read by the interviewer. Each participant was given a coupon for 500 JPY (approximately 5 USD) for completing all of the questionnaires in the Omnibus.

*Sampling: IQOS user survey*

Upon purchasing an *IQOS* device, consumers are offered the opportunity to register in the PMI Japan *IQOS* owner database, and, while doing so, participants can also agree to be contacted in the future for research purposes. As communicated by the Philip Morris Japan IQOS consumer database team, as of July 2017, 350,000 adult *IQOS* owners were registered in the PMI Japan *IQOS* owner database, which represented a coverage of approximately 45% of *IQOS* owners at the time (Philip Morris Japan Limited, written communication, February 2019).

Members of the PMI Japan *IQOS* owner database who agreed to be contacted for research purposes were selected randomly, contacted by email and invited for participation in each wave, taking into account the demographic distribution of the database (on the basis of age group and sex). Once the target sample size was reached within a wave, the survey was closed, and the respondents could not be sampled in a subsequent wave. The inclusion criteria were the same as those for the general adult population sample, with the addition that the adult participant had to have been a current daily or non-daily *IQOS* user in the past 30 days and consumed more than 100 *HeatSticks* (tobacco-containing sticks to be used with *IQOS*) in his/her lifetime. The *IQOS* users sample also completed the same “Tobacco Use Prevalence” questionnaire as the general population but as an online web survey.

*Ethical conduct of the study*

Prior to the start of the study, the study protocol and associated documents were reviewed and approved by the Hakata Clinic Institutional Review Board (Reference: P1-PMX-01-JP). In the general population survey, in line with the Omnibus processes, consent for participation in the general population Omnibus was given orally and recorded in a form by the interviewer; before answering the questions in the survey, the participants received an information sheet with background information on the research and were free to decline participation. For the *IQOS* user survey, electronic informed consent was obtained from all participants before they completed the survey online (i.e., participants had to indicate with a Yes/No response that they had read and understood the information about the study and agreed to participate in the survey). Overall, the study was performed in accordance with ethical principles that have their origin in the Declaration of Helsinki ^20^ and were consistent with Good Epidemiological Practice. ^21, 22^

### Study size

*General population survey*

The target sample size for each year was set at 5,000 participants. This number sufficed to estimate sampling error with a 95% confidence interval (CI) and precision of ±0.275 percent units given a population prevalence of *IQOS* use considered to be around 1%. ^23^ To achieve this sample size, four waves of 1,250 adult participants were sampled.

*IQOS user survey*

The target sample size for each year was set at 2,000 participants. This number sufficed to estimate with a 95% CI and a precision of ±2.19 percent units given a population percentage of *IQOS* use in combination with other TNP considered to be around 50%. ^23^ Each annual survey sample of *IQOS* users consisted of four waves, with the aim of recruiting 500 adult participants per wave.

### Analytical methods

Analyses were conducted using the SAS v9.4 software (SAS Inc., USA). Data were analyzed and summarized descriptively for Year 1 (combination of waves 1–4), and the prevalence of current use according to the type of product (cigarettes, *IQOS*, e-cigarettes, or other TNPs [including smokeless tobacco (chewing tobacco, snus, or snuff), smokeless tobacco pipe, cigars/pipes/kiseru/shisha, Ploom (tobacco vaporizer), and nicotine replacement therapy]) was calculated in the general population survey. Current use was defined as use of the product at the time of the survey and prior use of the product (daily or non-daily) in the past 30 days. Dual use was defined as current use of two types of TNP, and poly use was defined as current use of three or more types of TNP. Average daily consumption of TNP (mean consumption per day) refers to average number of units/times of product use per day on days used in the past 30 days. For participant characteristics, the summary statistics for continuous data included mean and standard deviation (SD). For categorical data, frequency and percentage were calculated. For all point estimates, 95% CI are reported. The prevalence of current use in the general adult population sample was also stratified and presented by age, sex, education level, and occupation status. The distribution of exclusive, dual, and poly use (patterns of use) and average daily consumption of TNP was determined among all current adult users in the *IQOS* user survey. An assessment of those who initiated TNP use when they started using *IQOS* in the 12 months prior to the survey was also carried out in both surveys.

For the general adult population survey, prevalence estimates were also standardized to the demographic structure (age and sex) of the Japanese population on the basis of the latest official Japanese census statistics. ^24, 25^ However, there were no major differences between the standardized (by age and sex) and unstandardized prevalence estimates because of quota application at the sampling stage; therefore, the unstandardized estimates are presented here.

## Results

### Sample characteristics

*General population survey*

**Figure 1.**
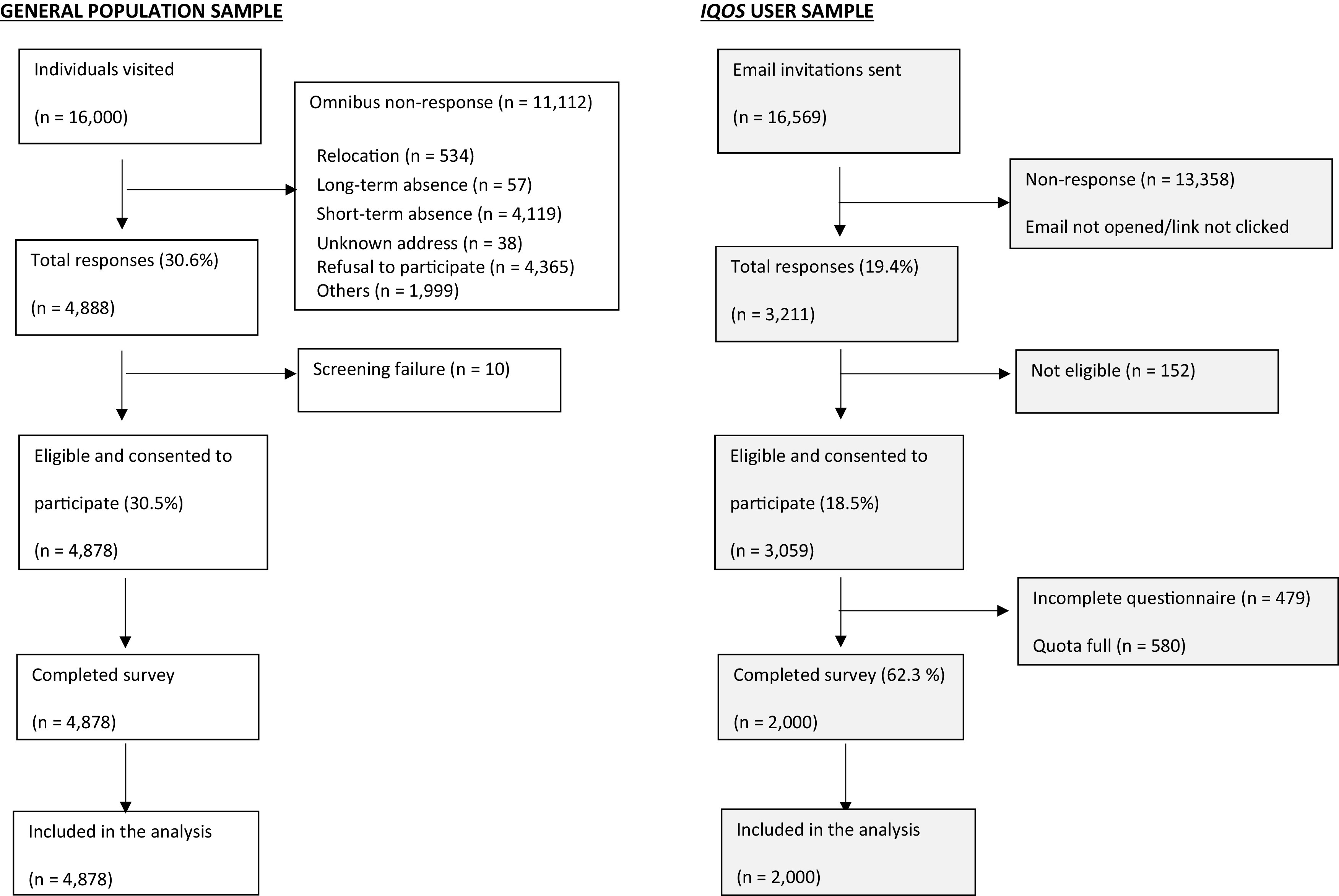
Flow diagram of study sample composition and response rate.

For the Omnibus, a response rate of 30% was achieved in the first year of the survey. This equates to 4,878 participants from a starting sample of 16,000 individuals. See Figure 1 for a breakdown of the participation rate. Table 1 presents the characteristics of the sample, as well as the Japanese population based on the most recent census data at the time of the survey. The demographic breakdown is, overall, comparable to that of the Japanese population. The age of the participants ranged from 20 to 97 years, with a mean (±SD) age of 53.7 (±17.92) years. While 48.1% of the participants were male, 51.9% were female. The highest level of education completed was high school in 49.1% of the sample and college or university education in 40.6%. The most common occupations were housewife (24.8%), manual employee (21.8%), and clerical employee (19.0%); and 17.1% of the participants were retired/unemployed.

**Table 1.** Sample characteristics of each survey and the distribution of the Japanese general population.

	Japanese population ^1^	General population survey (n = 4,878)		IQOS user survey (n = 2,000)	
	%	n	% [95% CI]	n	% [95% CI]
Sex					
Male	48.3	2,345	48.1 [46.6–49.5]	1,632	81.6 [79.8 *–*83.3]
Female	51.7	2,533	51.9 [50.5–53.4]	368	18.4 [16.7 *–*20.2]
*Age (mean) [SD]*			*53.75 [17.91]*		*38.50 [9.73]*
Age groups (years)					
20 *–*29	12.0	528	10.8 [9.9–11. 8]	420	21.0 [19.2 *–*22.9]
30 *–*39	15.1	723	14.8 [13.8–15.9]	736	36.8 [34.6 *–*39.0]
40 *–*49	17.8	873	17.9 [16.8–19.1]	568	28.4 [26.4 *–*30.5]
50+	55.2	2,754	56.5 [55.0–57.9]	276	13.8 [12.3 *–*15.4]
Education level					
Junior high school	8.6	454	9.3 [8.5 *–*10.2]	124	6.2 [5.1 *–*7.4]
High school	40.1	2,395	49.1 [47.6 *–*50.6]	726	36.3 [34.1 *–*38.5]
College/University	41.8	1,980	40.6 [39.2 *–*42.0]	1,135	56.8 [54.5 *–*59.0]
Don’t know/Not applicable	9.5	49	1.0 [0.7 *–*1.4]	15	0.8 [0.4 *–*1.3]
Occupation ^4^					
Farming/Agriculture/Fishery		80	1.6 [1.3 *–*2.1]	8	0.4 [0.1 *–*0.8]
Self-employed/Small private business	12.1 ^2^	538	11.0 [10.1 *–*12.0]	329	16.5 [14.8 *–*18.2]
Clerical employee		927	19.0 [17.9 *–*20.2]	284	14.2 [12.6 *–*15.9]
Manual employee		1,063	21.8 [20.6 *–*23.0]	268	13.4 [11.9 *–*15.0]
Managing profession	46.9 ^3^	118	2.4 [2.0 *–*2.9]	414	20.7 [18.9 *–*22.6]
Housewife	19.9	1,211	24.8 [23.6 *–*26.1]	84	4.2 [3.3 *–*5.2]
Student	2.0	106	2.2 [1.7 *–*2.7]	37	1.9 [1.3 *–*2.6]
Retired/Unemployed	19.1	835	17.1 [16.0 *–*18.3]	26	1.3 [0.8 *–*1.9]

Data (n = 4,878) are % or n (%) and 95% confidence intervals (CI).

^1^ Source: Statistics Bureau of Japan (2015) Source on Education: Statistics Bureau of Japan (2010) Source on Occupation: Public Opinion Survey on the Life of the People (23 June - 10 July 2016).

^2^ Self-employed individuals and those working in farming and agriculture.

^3^ Individuals in professional employment.

^4^ N=550 individuals in IQOS user sample reported ‘Don’t know/Not applicable‘ for Occupation category.

*IQOS user survey*

Overall, 16,569 email invitations were sent out in four waves in the first year of the survey; 3,211 individuals clicked on the survey link, representing a response rate of 19.4%. Within the four waves of the first year, 2,000 individuals completed the survey, representing a completion rate of 62.3%. See Figure 1 for a breakdown of the participation rate. Table 1 shows the characteristics of the *IQOS* user sample. The mean (±SD) age of the participants was 38.50 (±9.73) years. While 81.6% of the participants were male, 18.4% were female. The highest level of education completed was college or university education in 56.8% of the sample and high school in 36.3%. The participants were most commonly engaged in managing professions (20.7%) and self-employed/small private business (16.5%).

### Prevalence of TNP use in the general population survey

The overall prevalence (and 95% CI) of current use of any TNP was 18.5% (17.2–19.5%). As detailed in Table 2, the prevalence of current cigarette smoking was 17.5% (16.4–18.6%), while the prevalence of current *IQOS* use was 1.8% (1.4–2.2%; n = 86). The prevalence of current e-cigarette and other TNPs use were 0.7% (0.5–1.0%) and 1.8% (1.4–2.2%), respectively. Among the current TNP users (n = 887), 96.1% (94.5–97.3%) were cigarette smokers, 9.7% (7.8–11.9%) were *IQOS* users, and 3.9% (2.7–5.5%) were e-cigarettes users. In the general population sample, 1.3% (0.9–1.7%; n = 62) of individuals (i.e., 60% of all *IQOS* users) had started using *IQOS* within the past 12 months prior to the survey, and 96.8% (88.8–99.7%; N = 60) of these individuals had used other TNP prior to using *IQOS.*

**Table 2.** Prevalence of TNP use in the general population survey (overall and stratified by sample characteristics).

Characteristics	Prevalence n (%) [95% CI]			
	Cigarettes	*IQOS*	E-cigarettes	Other products *
Overall	852 (17.5) [16.4 *–*18.6]	86 (1.8) [1.4 *–*2.2]	35 (0.7) [0.5 *–*1.0]	86 (1.8) [1.4 *–*2.2]
Sex				
Male	654 (27.9) [26.1 *–*29.9]	70 (3.0) [2.3 *–*3.8]	25 (1.1) [0.6 *–*1.6]	66 (2.8) [2.1 *–*3.6]
Female	198 (7.8) [6.8 *–*9.0]	16 (0.6) [0.3 *–*1.1]	10 (0.4) [0.1 *–*0.8]	20 (0.8) [0.4 *–*1.3]
Age group (years)				
20 *–*29	98 (18.6) [15.3 *–*22.2]	20 (3.8) [2.3 *–*5.8]	9 (1.7) [0.7 *–*3.3]	7 (1.3) [0.5 *–*2.8]
30 *–*39	172 (23.9) [20.7 *–*27.2]	23 (3.2) [2.0 *–*4.8]	6 (0.8) [0.3 *–*1.9]	18 (2.5) [1.4 *–*4.0]
40 *–*49	214 (24.5) [21.7 *–*27.6]	25 (2.9) [1.8 *–*4.3]	9 (1.0) [0.4 *–*2.0]	26 (3.0) [1.9 *–*4.4]
50+	368 (13.4) [12.1 *–*14.8]	18 (0.7) [0.3 *–*1.1]	11 (0.4) [0.2 *–*0.8]	35 (1.3) [0.8 *–*1.8]
Education level				
Junior high school	79 (17.4) [14.0 *–*21.3]	5 (1.1) [0.3 *–*2.6]	5 (1.1) [0.3 *–*2.6]	9 (2.0) [0.9 *–*3.8]
High school	469 (19.6) [18.0 *–*21.3]	44 (1.8) [1.3 *–*2.5]	18 (0.8) [0.4 *–*1.2]	44 (1.8) [1.3 *–*2.5]
College/University	299 (15.1) [13.5 *–*16.8]	37 (1.9) [1.3 *–*2.6]	12 (0.6) [0.3 *–*1.1]	32 (1.6) [1.1 *–*2.3]
Don’t Know/Not applicable	5 (10.2) [3.3 *–*22.3]	0	0	1 (2.0) [0.0 *–*10.9]
Occupation				
Farming/Agriculture/Fishery	9 (11.5) [5.4 *–*20.8]	1 (1.3) [0.0 *–*6.8]	0	0
Self-employed/Small private business	156 (29.1) [25.2 *–*33.2]	15 (2.8) [1.5 *–*4.6]	7 (1.3) [0.5 *–*2.7]	17 (3.2) [1.8 *–*5.1]
Clerical employee	140 (15.1) [12.8 *–*17.6]	29 (3.1) [2.1 *–*4.5]	7 (0.8) [0.3 *–*1.6]	14 (1.5) [0.8 *–*2.6]
Manual employee	312 (29.4) [26.6 *–*32.3]	31 (2.9) [2.0 *–*4.2]	16 (1.5) [0.8 *–*2.5]	33 (3.1) [2.1 *–*4.4]
Managing profession	38 (32.2) [23.8 *–*41.5]	5 (4.3) [1.4 *–*9.7]	0	4 (3.4) [0.9 *–*8.5]
Housewife	58 (4.8) [3.6 *–*6.2]	4 (0.3) [0.0 *–*0.9]	4 (0.3) [0.0 *–*0.9]	6 (0.5) [0.1 *–*1.1]
Student	16 (15.1) [8.8 *–*23.4]	0	1 (0.9) [0.0 *–*5.2]	0
Retired/Unemployed	123 (14.7) [12.3 *–*17.4]	1 (0.1) [0.0 *–*0.7]	0	12 (1.4) [0.7 *–*2.5]

Data (n = 4,878) are n (%) and 95% confidence intervals (CI).

Cigarettes: Manufactured and roll-your-own cigarettes; *IQOS*: *IQOS* with *HeatSticks*; TNP: tobacco and nicotine-containing products.

* Other products: including smokeless tobacco (chewing tobacco, snus, or snuff), smokeless tobacco pipe, cigars/pipes/kiseru/shisha, Ploom (tobacco vaporizer), and nicotine replacement therapy.

The prevalence (and 95% CI) of cigarette smoking was 27.9% (26.1–29.9%) in men and 7.8% (6.8–9.0%) in women. For *IQOS* use, the prevalence in men and women were 3.0% (2.3–3.8%) and 0.6% (0.3–1.1%), respectively. The prevalence of cigarette smoking was the highest in the 40–49 age group, followed by the 30–39 years age group, and lowest in the 50+ age group (13.4% [12.1–14.8%]). The prevalence of *IQOS*, e-cigarette, and other TNPs use was similarly distributed across all age groups but tended to be lower in the 50+ age group.

Cigarette smoking was most prevalent in participants who had completed high school-level education (19.6% [95% CI: 18.0–21.3%]). In contrast, *IQOS* use was most prevalent among those who had completed college/university-level education (1.9% [1.3–2.6%]), followed by those who had completed high school-level education [1.8% (1.3–2.5%)]. The prevalence (and 95% CI) of cigarette smoking and *IQOS* use were also the highest among individuals in a managing profession (32.2% [23.8–41.5%] and 4.3% [1.4–9.7%], respectively).

### Distribution of use patterns among current TNP users in the general population and *IQOS* user survey

The patterns of current exclusive, dual, and poly product use among current TNP users in the general population survey are presented in Table 3. Of those participants for whom patterns could be defined (n=876), the majority 80.5% [95% CI: 77.6–83.1%] were only smoking cigarettes, whereas 2.5% [1.5–3.8%] were only using *IQOS*. The most common dual use patterns were cigarettes combined with other products (excluding *IQOS* and e-cigarettes) (7.3% [5.6–9.3%]), cigarettes and *IQOS* (4.6% [3.2–6.2%]), and cigarettes and e-cigarettes (1.5% [0.7–2.6%]). For poly-use (i.e., use of three or more products), the most common combination was cigarettes, *IQOS*, and e-cigarettes in 1.1% [0.5–2.1%] of current TNP users.

**Table 3.** Distribution of patterns of use among current TNP users (n=876) in the general population survey.

	Number (n)	Percentage [95% CI]
Exclusive users		
Cigarettes	705	80.5 [77.6–83.1]
*IQOS*	22	2.5 [1.5–3.8]
E-cigarettes	3	0.3 [0.0–1.0]
Dual users		
Cigarettes + *IQOS*	40	4.6 [3.2–6.2]
Cigarettes + e-cigarette	13	1.5 [0.7–2.6]
*IQOS* + e-cigarette	5	0.6 [0.1–1.4]
Cigarettes + other product *	64	7.3 [5.6–9.3]
*IQOS* + other product *	4	0.5 [0.1–1.2]
E–cigarette + other product *	1	0.1 [0.0–0.7]
Poly users		
Cigarettes + *IQOS* + e-cigarette	10	1.1 [0.5–2.1]
Cigarettes + other products *	2	0.2 [0.0–0.9]
Cigarettes + *IQOS* + other products *	4	0.5 [0.1–1.2]
Cigarettes + e-cigarette + other products *	3	0.3 [0.0–1.0]

Cigarettes: combustible cigarettes including Manufactured and roll-your-own cigarettes.

* Other product(s): including smokeless tobacco (chewing tobacco, snus, or snuff), smokeless tobacco pipe, cigars/pipes/kiseru/shisha, Ploom (tobacco vaporizer), and nicotine replacement therapy.

Exclusive users: current use of only one type of TNP; Dual users: current use of two types of TNP; Poly users: Current use of three or more types of TNP.

The patterns of current exclusive, dual, and poly product use in the *IQOS* user sample are presented in Table 4. Of the participants in the *IQOS* user survey, 74.8% had started using the product within the 12 months prior to the survey, and 98% of these *IQOS* users had been using other TNP prior to using *IQOS.* Of those participants for whom patterns could be defined, 63.4% [61.2–65.6%] were exclusive users of *IQOS.* The most common dual use pattern was *IQOS* combined with cigarettes (20.6% [18.7–22.5%]), and, among poly users, the most common combination was *IQOS*, cigarettes, and other TNPs (7.3% [6.1–8.6%]). On average, exclusive *IQOS* users used the product 29.6 [29.4–29.8] days out of the past 30 days and on days used, the average daily consumption of *IQOS HeatSticks* was greater among exclusive *IQOS* users (16.8 *HeatSticks*) than among dual and poly users, who used 13.8 and 12.8 *HeatSticks*, respectively. The average daily consumption of cigarettes was 11.0 among dual cigarette and *IQOS* users and 12.8 among poly users.

**Table 4.** Distribution of patterns of use and consumption in the IQOS user survey.

Patterns of use	Number (n)	Percentage [95% CI]	Mean number of days used in past 30 days [95% CI]		Mean consumption per day [95% CI]	
			Cigarettes	*IQOS* ( *HeatSticks*)	Cigarettes	*IQOS* ( *HeatSticks*)
Exclusive users						
*IQOS*	1,234	63.4 [61.2–65.6]		29.6 [29.4–29.8]		16.8 [16.3–17.3]
Dual users						
*IQOS*–CC	400	20.6 [18.7–22.5]	23.8 [22.8–24.9]	28.1 [27.5–28.6]	11.0 [10.1–12.0]	13.8 [12.9–14.6]
*IQOS*–E-cigarettes	34	1.7 [1.2–2.5]	-	29.2 [27.5–30.9]		16.7 [14.1–19.3]
*IQOS*-other products ^1^	96	4.9 [4.0–6.0]	-	29.4 [28.8–30.0]		17.9 [16.2–19.5]
Poly users	182		24.7 [23.0–26.3]	27.2 [26.3–28.1]	14.3 [12.8–15.9]	14.1 [12.9-15.4]
*IQOS*–CC–other products ^1^	142	7.3 [6.1–8.6]			12.8 [11.1–14.5]	12.8 [11.3–14.2]
*IQOS*–other products ^1^ (poly use without cigarettes)	40	2.1 [1.4–2.8]				14.6 [12.2–17.0]

^1^ Other products: including smokeless tobacco (chewing tobacco, snus, or snuff), smokeless tobacco pipe, cigars/pipes/kiseru/shisha, Ploom (tobacco vaporizer), and nicotine replacement therapy; CC: Manufactured and roll-your-own cigarettes.

## Discussion

Three years after the launch of *IQOS* in Japan in 2014, the prevalence of current *IQOS* use in 2017 was 1.8% in the general population survey. The prevalence of cigarette smoking in the present study (17.5%) is in line with the reported prevalence of 18.2% in the Japan National Health and Nutrition Survey in 2015. ^11, 26^ However, at the time of this study, this national survey did not report the prevalence of use of other smoke-free TNP, such as *IQOS* and only in 2018 did this national survey report a 5.1% prevalence of heated tobacco products in general. Adamson *et al.* ^15^ also reported a 5% prevalence of heated tobacco product use in their regional cross-sectional study, which is higher than the prevalence observed in the present study. However, they also included other heated tobacco products (glo and Ploom TECH) in their prevalence count, and alike the Japan National Health and Nutrition Survey, their data were collected in 2018, a year after the present study. Additional data, from the 2018 ITC Japan nationally representative web survey of 4684 participants by Sutanto *et al.*, ^27^ similarly show low prevalence (2.7%) of current heated tobacco product use, and even lower prevalence (0.9%) of exclusive heated tobacco product use with most users also smoking cigarettes. It is feasible that there had been an increase in the use of these products by 2018. The increasing use of heated tobacco products was reported by Tabuchi *et al*., ^13^ who reported that the prevalence of current use of *IQOS* increased from 0.3% in 2015 to 0.6% in 2016 and up to 3.6% in 2017. Like Adamson *et al.,* these authors also reported a higher prevalence than that in the present study; however, their online non-representative survey included mostly a younger sample from the Japanese adult population. For instance, in their sample, 64.3% of the participants were below 50 years of age, while only 43.5% of the general population sample in the present study were below 50 years of age (similar to the reported 2015 census data of 44.8%). ^25^ Adamson *et al.* similarly oversampled participants in the younger 20-24 year age group. Nonetheless, the samples from these studies are more reflective of our *IQOS* user survey sample, which was also younger, on average. Together, these findings seem to support the fact that, similar to cigarette smoking, *IQOS* use tends to be more prevalent among men and middle-aged adults. However, unlike cigarette smoking, which is most prevalent among individuals with lower educational attainment, ^28^ *IQOS* use was more prevalent among individuals with higher educational attainment and in white-collar professions. These characteristics also tend to typify early adopters of a new product or technology such as *IQOS* and could change as more smokers in the population adopt *IQOS*.

Notably, our findings show low initiation of TNP use with *IQOS*; the majority of *IQOS* users in both the general adult population (96.8%) and *IQOS* user (98%) surveys had been using other TNP prior to using *IQOS.* This supports the findings of other studies, ^13, 14, 29, 30^ as well as the overall goal of tobacco harm reduction, that the majority of current *IQOS* users are the intended users for such potential reduced-risk products ^2^ (i.e., adult cigarette smokers switching to the product instead of continuing smoking and low initiation and re-initiation of TNPs by never users or former users, respectively).

The results of the present surveys indicate a rapid expansion of *IQOS* in the Japanese market in 2017; 60% of *IQOS* users in the general adult population and 74.8% of participants in the *IQOS* user survey had started using the product within the 12 months prior to the survey. Hence, it is conceivable that, for early adopters of the products, complete displacement of cigarettes might take time, and, for some smokers, a period of dual use may be expected prior to exclusive *IQOS* use. ^31, 32^ In this regard, the present results showed that 36.6% of participants in the *IQOS* user sample survey used the product in combination with other TNP, whereby 20.6% reported dual use with cigarettes. Patterns of dual and poly product use were also observed in the general population sample. Subsequent repeated cross-sectional data are needed to closely monitor the trends in multiple product use as *IQOS* use increases within a population and its implications for tobacco harm reduction.

Some limitations of the present study should be considered. First, the study relied only on self-reported product use. Previous studies have shown that the reliability of self-reported smoking in adults is generally high, suggesting that self-reported data provide reasonably valid estimates of cigarette smoking in the population. ^33, 34^ However, the reliability of self-reported assessments has not yet been investigated and confirmed for smoke-free TNPs to the same extent and might warrant further investigation. Second, compared with other nationwide Japanese population surveys ^11^ that achieved response rates of 50–60%, the Omnibus had a relatively low response rate (30.5%). Overall, while the response rates to population surveys in Japan have been on the decline in recent years, this decline might also be influenced by the sampling methods employed. ^35^ Other surveys ^36^ tend to employ multimodal household, mail, telephone, and web-based methods for recruitment and data collection to improve response rates, whereas the Omnibus solely used a door-to-door household approach. However, the added value of an Omnibus survey is that it covers a range of topics and not only tobacco use behavior, which encourages people’s willingness to participate in the survey. Moreover, the current survey also provided the respondents with a self-administration option for added privacy. Subsequently, a particular non-response bias toward the items of the current survey was not evident; only 10 of the 4,888 eligible participants from the Omnibus did not consent to completing the specific “Tobacco Use Prevalence” questionnaire. Nevertheless, the overall low response (19.4%) and completion (62.3%) rates for the web survey in the *IQOS* user sample could perhaps have been improved through multi-modal methodological changes in survey distribution, use of follow-up reminders, or greater incentives for participating in the web survey. Third, *IQOS* was newly introduced on the market at the time of the survey and the decision to adopt an innovation such as switching from cigarette smoking to sustained exclusive use of products such as *IQOS* is a dynamic process, with interactions among individual, situational, and contextual factors as well as attributes of the innovation itself. ^19^ Therefore, the present study can only provide some insight into some characteristics of the early adopters (e.g., demographic information such as age, sex, education, and career status). The longer the product is on the market, further investigations on what contributes to the *IQOS* adoption process will also need to consider the intrinsic and extrinsic attributes of the product itself (e.g., its relative advantage and compatibility within the adult population), the impact of tobacco regulatory policies, including on minors and youths, and other behavioral and environmental factors especially as more individuals adopt the use of *IQOS*. Lastly, the findings from the *IQOS* user sample are from a consumer panel which may not be representative of all *IQOS* users since the panelists are self‐selected and incentivized to participate in follow‐up surveys. Nevertheless, a substantial portion of estimated number of *IQOS* users in Japan are registered in this database and hence it provides access to a large number of *IQOS* users and more accurate picture of the use patterns of *IQOS* users which would not be possible with the sampling strategy of the general population given the low prevalence of *IQOS* use in the general population at the time of the survey. Future surveys should consider larger sample sizes in the general population surveys to allow for precise estimation of *IQOS* use patterns with other TNPs.

The main strength of the study lies in its design: annual repeated collection of data by using the same sampling framework and methods—namely face-to-face interviews of a national representative sample of participants coupled with a web survey of a large *IQOS* user sample—for gaining complimentary insights into a sizeable number of early adopters of the product. With additional waves and annual data, the present survey will continue to provide insights into the trends in prevalence and adoption of *IQOS* use in the Japanese adult population.

## Conclusion

As of 2017, cigarette smoking remains the most prevalent way of consuming TNP in Japan; however, alternatives such as *IQOS* are already being adopted by a large number of adult Japanese smokers, with low initiation among TNP never users and low re-initiation by former TNP users. This suggests the potential of smoke-free TNP as a harm-reduction alternative for replacing cigarettes. The present results complement those of previous surveys on the prevalence and use patterns of different TNP and serve as baseline data for monitoring early uptake and population trends over time in the use of potential smoke-free TNP in Japan.

## Data availability

### Underlying data

INTERVALS: YEAR 1 DATA (SAS DATASETS, CC-BY), https://doi.org/10.26126/intervals.vb4omj. ^37^

This project contains the following underlying data:

• Three SAS datafiles in the Clinical Data Interchange Standards Consortium (CDISC) Analysis Data Model (ADaM) structure ( www.cdisc.org/standards).
  ◦ The ADSL (adsl.sas7bdat) is the Subject Level Analysis Dataset and contains the main information on participants identifier, demographics, and tobacco and/or nicotine product use groups and patterns to facilitate analysis and interpretation of analysis.
  ◦ ADQS (adqs.sas7bdat) is the Questionnaire Analysis Dataset and contains specific information on the study survey, i.e. all questions and items answered by participants in the survey.
  ◦ ADEX (adex.sas7bdat) is the Exposure Analysis Dataset and contains specific information on the tobacco and/nicotine product use exposure, i.e. all questions and items answered by participants in the survey related to their product use.
  ◦ The ADAM specification and metadata file (ADaM_PMX01JP_AnY1_DBconv.xlsx) contains the dataset and variable labels and definitions, code lists to decode the variables names, terms and values, and the methods and computational algorithms to derive the analytical datasets.

### Extended data

INTERVALS: YEAR 1 DATA (SAS DATASETS, CC-BY), https://doi.org/10.26126/intervals.vb4omj. ^37^

This project contains the following extended data:

• “Tobacco Use Prevalence” questionnaire (Year 1 Tobacco Use Prevalence Questionnaire_eng_jp.pdf) is the questionnaire administered in the general population and IQOS user survey (Japanese and English version).

Data are available under the terms of the Creative Commons Attribution 4.0 International license (CC-BY 4.0).
