# Supplementary material for: Prevalence and patterns of tobacco and/or nicotine product use in Japan (2017) after the launch of a heated tobacco product ( IQOS ®): a cross-sectional study
Source: F1000Res. 2022 Mar 21;10:504. Originally published 2021 Jun 25. [Version 2] doi: 10.12688/f1000research.52407.2 (PMC9069173; doi:10.12688/f1000research.52407.2)
Supplement: Peer review history

# Peer review: Reviewer response for version 2

**DOI:** 10.5256/f1000research.122553.r128142

**Published:** 2022-05-04

**Authors:** Kenneth Michael Cummings

**Affiliations:** 1 Department of Psychiatry & Behavioral Sciences, Hollings Cancer Center, Medical University of South Carolina, Charleston, SC, USA

I appreciate the authors response to prior comments. 

 The addition of Table 3 to the paper is important and the findings despite small numbers should highlighted in the abstract. The predominate pattern of use of IQOS in the community sample was dual/poly use with cigarettes (59/85 = 69%). That is important to acknowledge, even with the caveat of low user numbers in absolute terms. Since this paper is now recast as a baseline upon with to compare the growth of IQOS and other heat tobacco products in Japan it is important to establish the low prevalence/high dual use pattern shown initially. 

 One other suggestion is to report the number of exclusive other product users in table 3. I would also provide a breakdown of other products used since a number of products are listed in the footnote, but given the descriptive nature of the study actually reporting the products reported would be helpful, especially since non-IQOS heat tobacco products were included in the other category.

Is the work clearly and accurately presented and does it cite the current literature?

Partly

If applicable, is the statistical analysis and its interpretation appropriate?

Partly

Are all the source data underlying the results available to ensure full reproducibility?

No

Is the study design appropriate and is the work technically sound?

Partly

Are the conclusions drawn adequately supported by the results?

Yes

Are sufficient details of methods and analysis provided to allow replication by others?

Partly

Reviewer Expertise:

I'm interested in products and policies that will accelerate a decline in smoking related diseases. I've spent 40 years doing research on the harms of tobacco, smoking prevention and cessation. I have also followed closely the history of tobacco product innovations such as filters, low tar/light cigarettes and their failure to reduce the harms caused by cigarettes.

I confirm that I have read this submission and believe that I have an appropriate level of expertise to confirm that it is of an acceptable scientific standard.

# Peer review: Reviewer response for version 2

**DOI:** 10.5256/f1000research.122553.r128143

**Published:** 2022-04-26

**Authors:** Lai Wei

**Affiliations:** 1 Center for Research & Technology, Altria Client Services LLC, Richmond, VA, USA

The authors have addressed all my comments. The additions of Table 3 (pattern of use from the general population survey) and Table 4 (with added cigarette use frequency and consumption) provide valuable insights to further understand IQOS use behavior in Japan.

 I have a few minor suggestions:

1. In the abstract (first sentence) and introduction (first paragraph), the term ‘TNP’ was introduced as ‘smoke-free tobacco and/or nicotine-containing products. The reviewer believes the term ‘TNP’ is referring to ‘Tobacco and/or Nicotine Products’ in some of the content. Examples include “assessed the prevalence of current TNP use according to type of product (cigarettes, IQOS, e-cigarettes, and other TNPs)”, “The prevalence of current use across all TNP in the sampled general population was 18.5%, with 17.5% for cigarette smoking and 1.8% for IQOS use”, etc. The reviewer suggests clarifying this ‘TNP’ term, going through the article to examine each use case of ‘TNP’.
2. In discussion of Table 4 results, the reviewer suggests reiterating the sampling differences between the general population survey and the IQOS user survey. The participants in the IQOS user survey must have been consumed more than 100 HeatSticks in his/her lifetime. This inclusion criteria excluded IQOS experimental users from the IQOS user survey, which lead to the differences in the proportions of exclusive and dual/poly use when making comparisons between the two surveys. If data exists, it would add value to report the proportion of having consumed more than 100 HeatSticks in his/her lifetime among current IQOS users.
3.  In Table 4, cigarette consumption measures for poly users should be moved down to the row ‘IQOS-CC-other products’. And for consistency, a dash ‘-‘ sign should be added in places where cigarette consumption measures are not applicable.

Is the work clearly and accurately presented and does it cite the current literature?

Yes

If applicable, is the statistical analysis and its interpretation appropriate?

Yes

Are all the source data underlying the results available to ensure full reproducibility?

Yes

Is the study design appropriate and is the work technically sound?

Yes

Are the conclusions drawn adequately supported by the results?

Yes

Are sufficient details of methods and analysis provided to allow replication by others?

Yes

Reviewer Expertise:

Statistics, Epidemiology, Population Modeling and Tobacco Survey Research.

I confirm that I have read this submission and believe that I have an appropriate level of expertise to confirm that it is of an acceptable scientific standard.

# Peer review: Reviewer response for version 1

**DOI:** 10.5256/f1000research.55681.r98493

**Published:** 2021-11-09

**Authors:** Kenneth Michael Cummings

**Affiliations:** 1 Department of Psychiatry & Behavioral Sciences, Hollings Cancer Center, Medical University of South Carolina, Charleston, SC, USA

This is an interesting paper authored by Philip Morris scientists. The paper presents descriptive data on the prevalence of heat tobacco product use among adults from the 2016 and 2017 online Omnibus cross-sectional surveys which are designed to be representative the adult population in Japan. Analyses are based on 4,878 survey responders out of 16,000 households approached to participate. It appears the authors have combined results from the 2016 and 2017 Omnibus surveys which is unfortunate, since it prevents readers from seeing trends in the use of heated tobacco products overtime, as well as changes in cigarette smoking (potential displacement of cigarette use). A third survey conducted in 2018 is mentioned in the paper, but is not included in the analysis. It should be. The study also presents data on 2000 person customer IQOS user survey. Unfortunately, the source of this survey is not well described in the paper. The findings from the Omnibus surveys show that the predominate tobacco product consumed ay adults is cigarettes at 17.5% vs 4.0% for heated tobacco products and e-cigarettes combined. However, these prevalence figures (derived) from table 2 are likely inflated since it is likely that there is dual use across the product categories listed in the table. Unfortunately, the authors do not talk about dual use from their population based omnibus surveys, but instead report on dual use from their non-presentative consumer tracking study were dual use is likely to be lower due to self section and incentives to participate in the consumer use follow-up surveys. I have two strong recommendations to the authors.

 First, they should report their population survey data separate from their consumer use surveys. Reader can get easily confused when these results based on very different groups are presented in the same paper. 

 Second, the most relevant findings for estimating smoking harm reduction are the findings from the population based surveys. The authors should present data separately for each survey year. Showing data for 2016 separately from 2017 and 2018 (and perhaps more recent surveys) would allow the reader to see trends in the use of heated tobacco products and different brands of products. Also, the authors should report on exclusive and dual use of products. I would suspect that many of those experimenting with heated tobacco products might dual use different brands. One of the main limitation of the paper as presented was the failure to present detailed data on dual, patterns of use, and duration of use of different tobacco products. The low number of heated tobacco users in each survey year is likely a reason for not sharing such details, but they should be available to readers with appropriate caveats about sample size. Also, the sample size problem is a fixable since Philip Morris could could implement a larger survey in each year so that they can effectively estimate low level use of IQOS and other heated tobacco products. The Omnibus survey results as presented are helpful as a preliminary level to assess prevalence so that a larger study could be undertaken to look a dual use, patterns of use, and duration of use of heated tobacco products. Also, a larger study would allow the authors to estimate more precisely the uptake and continued regular use of heated tobacco products by non-smokers. The current study showed low current use of heated tobacco products (<2%, and ~10% of overall nicotine/tobacco use). On a positive note, among those reporting starting the use of a heated tobacco product in the past 12 months, only 2 out of 62 (~3%) reported themselves to be non-smokers. However, this study was restricted to adults aged 21 and older so it is unclear from this study whether heated tobacco products are gaining attention among teenagers and young adults, similar to what has been observed for e-cigarettes in some countries. This is a limitation that needs to be highlighted in the discussion also the authors should comment on other data sources to estimate use of heated tobacco use by minors, since that is a area of public health concern, which can not be ignored. 

 I would encourage PMI to make their deidentified data from these surveys available online for other researchers to interrogate. Similar data sets available from other countries should also be make available. I would encourage other manufacturers to do the same since the data on prevalence of use, dual use and patterns of use are essential elements to modeling the potential longer term impact of heated tobacco products on population health. 

 The authors should also comment on the overall low use of IQOS in their study (1.8% prevalence overall). Other heat tobacco products introduced into Japan after IQOS had the same prevalence rate of 1.8% which was surprising since IQOS was introduced earlier than the competitor products. ENDS products which are banned for sale in Japan had a lower prevalence of 0.7%, which makes sense. It would be useful for the authors to include a table comparing the prevalence rates from this study to those from other published source. It was hard to follow the comparisons form the text. Other population studies in Japan have found high rates of dual use of cigarettes and heat tobacco products (see, for example, an article I was involved with: Sutanto E, *et al.* 2019 ^1^); it would be useful (essential) for the authors to report dual use data from their population surveys versus those from other surveys in Japan. Dual vs exclusive use of IQOS will be one of the key metrics researchers will be interested in evaluating.

Is the work clearly and accurately presented and does it cite the current literature?

Partly

If applicable, is the statistical analysis and its interpretation appropriate?

Partly

Are all the source data underlying the results available to ensure full reproducibility?

No

Is the study design appropriate and is the work technically sound?

Partly

Are the conclusions drawn adequately supported by the results?

Yes

Are sufficient details of methods and analysis provided to allow replication by others?

Partly

Reviewer Expertise:

I'm interested in products and policies that will accelerate a decline in smoking related diseases. I've spent 40 years doing research on the harms of tobacco, smoking prevention and cessation. I have also followed closely the history of tobacco product innovations such as filters, low tar/light cigarettes and their failure to reduce the harms caused by cigarettes.

I confirm that I have read this submission and believe that I have an appropriate level of expertise to state that I do not consider it to be of an acceptable scientific standard, for reasons outlined above.

## References

[1] : Prevalence, Use Behaviors, and Preferences among Users of Heated Tobacco Products: Findings from the 2018 ITC Japan Survey. *International Journal of Environmental Research and Public Health* .2019;16(23) : 10.3390/ijerph16234630 10.3390/ijerph16234630 PMC692680931766410

# Response

**Published:** 2022-03-16

**Authors:** Esther Afolalu

**Affiliations:** PMI R&D, Switzerland

This is an interesting paper authored by Philip Morris scientists. The paper presents descriptive data on the prevalence of heat tobacco product use among adults from the 2016 and 2017 online Omnibus cross-sectional surveys which are designed to be representative the adult population in Japan. Analyses are based on 4,878 survey responders out of 16,000 households approached to participate. It appears the authors have combined results from the 2016 and 2017 Omnibus surveys which is unfortunate, since it prevents readers from seeing trends in the use of heated tobacco products overtime, as well as changes in cigarette smoking (potential displacement of cigarette use). A third survey conducted in 2018 is mentioned in the paper, but is not included in the analysis. It should be.

***Thanks for this observation. The annual survey/annual sampling of the surveys do not correspond to a calendar year but rather consisted of four approximately equal-sized waves of participants spaced throughout the year to account for potential seasonal differences. The data presented in the paper are from repeated cross‐sectional surveys conducted in 2016 (wave 1 in December) and 2017 (waves 2, 3, and 4 in March, May, and July respectively). The Study design and Settings in Methods section are made clearer to reflect this. Another paper is in preparation to show the trends for subsequent years (Year 2 (2017-2018) and Year 3 (2018-2019) (see further below).***

 The study also presents data on 2000 person customer IQOS user survey. Unfortunately, the source of this survey is not well described in the paper.

***Upon purchasing an IQOS device, consumers are offered the opportunity to register in the PMI Japan IQOS owner database, and, while doing so, participants can also agree to be contacted in the future for research purposes. It was described in the section “Sampling: IQOS user survey” that these participants were members of the PMI Japan IQOS owner database who agreed to be contacted for research purposes and were selected randomly and contacted by email and invited for participation in each wave of the survey.***

 The findings from the Omnibus surveys show that the predominate tobacco product consumed ay adults is cigarettes at 17.5% vs 4.0% for heated tobacco products and e-cigarettes combined. However, these prevalence figures (derived) from table 2 are likely inflated since it is likely that there is dual use across the product categories listed in the table. Unfortunately, the authors do not talk about dual use from their population based omnibus surveys, but instead report on dual use from their non-presentative consumer tracking study were dual use is likely to be lower due to self section and incentives to participate in the consumer use follow-up surveys. I have two strong recommendations to the authors.

 First, they should report their population survey data separate from their consumer use surveys. Reader can get easily confused when these results based on very different groups are presented in the same paper. 

***Thank you for these suggestions.***

***We have now added an additional Table (Table 3) to show distributions of patterns of TNP use among current TNP users in the general population sample. However, as the results show, the prevalence of IQOS use in general population was expected and indeed seen to be quite low (N=85) which limits the extent to gather interpretable and detailed information on specific product use patterns such as dual/poly use. This is why we supplement this information with information in Table 4 on patterns of use of IQOS users from the consumer survey, and report this in the same paper to provide two complementary views.***

***Related to the comments that the data on dual use from the consumer panel is not representative because the panelists are self‐selected and have incentives to participate in follow‐up surveys, we acknowledge this limitation and have expanded on this in the Discussion section. Indeed, IQOS users are invited to join the IQOS owner database and receive incentives for completing the surveys. However, a substantial portion of estimated number of IQOS users in Japan are registered in this database and hence it provides access to a larger number of IQOS users and more precise and accurate picture of the use patterns of IQOS users which would not be possible with the sampling strategy of the general population given the rather low prevalence of IQOS use in the general population at the time of the survey. Participants from the database were randomly invited to participate in the survey based a quota that reflects the gender and age distribution of the population of users.***

 Second, the most relevant findings for estimating smoking harm reduction are the findings from the population based surveys. The authors should present data separately for each survey year. Showing data for 2016 separately from 2017 and 2018 (and perhaps more recent surveys) would allow the reader to see trends in the use of heated tobacco products and different brands of products.

 Also, the authors should report on exclusive and dual use of products. I would suspect that many of those experimenting with heated tobacco products might dual use different brands. One of the main limitation of the paper as presented was the failure to present detailed data on dual, patterns of use, and duration of use of different tobacco products. The low number of heated tobacco users in each survey year is likely a reason for not sharing such details, but they should be available to readers with appropriate caveats about sample size.

***This paper reflects the first year of this survey (Year 1 (2016-2017)) as an initial presentation of the data, another paper is in preparation to show the trends for subsequent years (Year 2 (2017-2018) and Year 3 (2018-2019)).***

***As explained above, we have now added an additional Table (Table 3) to show distributions of patterns of TNP use among current TNP users in the general population sample with the caveat about sample size and low prevalence of IQOS use in the general population at the time of the Year 1 survey.***

 Also, the sample size problem is a fixable since Philip Morris could could implement a larger survey in each year so that they can effectively estimate low level use of IQOS and other heated tobacco products. The Omnibus survey results as presented are helpful as a preliminary level to assess prevalence so that a larger study could be undertaken to look a dual use, patterns of use, and duration of use of heated tobacco products. Also, a larger study would allow the authors to estimate more precisely the uptake and continued regular use of heated tobacco products by non-smokers.

***Thank you for this key suggestion, this has been considered for ongoing and future surveys and this point mentioned as part of limitations in the Discussion section.***

 The current study showed low current use of heated tobacco products (<2%, and ~10% of overall nicotine/tobacco use). On a positive note, among those reporting starting the use of a heated tobacco product in the past 12 months, only 2 out of 62 (~3%) reported themselves to be non-smokers. However, this study was restricted to adults aged 21 and older so it is unclear from this study whether heated tobacco products are gaining attention among teenagers and young adults, similar to what has been observed for e-cigarettes in some countries. This is a limitation that needs to be highlighted in the discussion also the authors should comment on other data sources to estimate use of heated tobacco use by minors, since that is a area of public health concern, which can not be ignored. 

***Thank you for this suggestion. As this study was conducted in Japan, the lower age limit was 20 years old which represent the legal age to be authorized to buy tobacco products in Japan, and not 21 years old as stated. Additionally, we did not conduct the study in underage populations, hence it is beyond the scope of this paper to compare data sources on use by minors, however, we have included in the Discussion this should be a focus of further studies.***

 I would encourage PMI to make their deidentified data from these surveys available online for other researchers to interrogate. Similar data sets available from other countries should also be make available. I would encourage other manufacturers to do the same since the data on prevalence of use, dual use and patterns of use are essential elements to modeling the potential longer term impact of heated tobacco products on population health. 

***The deidentified Year 1 data reported in this paper is made publicly available, the link is in the data availability section of the manuscript https://doi.org/10.26126/intervals.vb4omj alongside high‐level study description: https://www.intervals.science/studies/#/htp‐use‐prevalence‐use‐patterns‐japan. As data from other years are published, the data will also be made publicly available.***

 The authors should also comment on the overall low use of IQOS in their study (1.8% prevalence overall). Other heat tobacco products introduced into Japan after IQOS had the same prevalence rate of 1.8% which was surprising since IQOS was introduced earlier than the competitor products. ENDS products which are banned for sale in Japan had a lower prevalence of 0.7%, which makes sense. It would be useful for the authors to include a table comparing the prevalence rates from this study to those from other published source. It was hard to follow the comparisons form the text.

***The low IQOS use prevalence reflects the period of the current survey (December 2016 – July 2017), which is soon after the national introduction of IQOS in Japan. We presented comparisons with similar surveys in the text and not a table in order to provide sufficient context and ensure there is no misunderstanding in the interpretation of the findings given the considerable differences with the other surveys such as differences in sampling strategies, use of a non-representative general population sample, reporting of heated tobacco products in general and not IQOS specifically, and surveys conducted later than the time period reported in current paper, etc...***

 Other population studies in Japan have found high rates of dual use of cigarettes and heat tobacco products (see, for example, an article I was involved with: Sutanto E, *et al.* 2019 ^1^ ); it would be useful (essential) for the authors to report dual use data from their population surveys versus those from other surveys in Japan. Dual vs exclusive use of IQOS will be one of the key metrics researchers will be interested in evaluating.

***In addition to the comparisons with other surveys described above, we have also now referred in the discussion to the Sutanto et al 2019 paper. As explained above, we have also now added an additional Table (Table 3) to show distributions of patterns of TNP use among current users in the general population sample with the caveat about sample size and low prevalence of IQOS use in the general population at the time of the Year 1 survey.***

# Peer review: Reviewer response for version 1

**DOI:** 10.5256/f1000research.55681.r88350

**Published:** 2021-07-13

**Authors:** Lai Wei

**Affiliations:** 1 Center for Research & Technology, Altria Client Services LLC, Richmond, VA, USA

The authors presented the prevalence and patterns of tobacco and/or nicotine-containing products (TNP) usage based on first annual (2016-2017) data of two cross-sectional surveys in Japan. The survey methodology and sampling methods were clearly described. The analysis results provided insights of the early adoption and market expansion of IQOS in Japan. The reviewer has listed the comments for each section below. The reviewer has the suggestions to add analysis results regarding exclusive, dual and poly use patterns from both surveys (i.e. general population survey and IQOS user survey) and the cigarette and IQOS use patterns (e.g. daily use, number of days used) for exclusive and dual/poly users.

**Abstract**

- The authors described that the analysis is based on ‘general adult population’ (and also mentioned ‘legal age’ in the Introduction section) while the analysis results were presented for 20 years of age or older. The authors should specify the age range of the baseline population upfront in the abstract as well as in the introduction or study design section as the ‘adult age’ or ‘legal age’ may differ by countries.

**Introduction**

- The authors stated ‘In the full sample, 16% reported exclusive use of heated tobacco products and 11% reported dual use with cigarettes’, following the mention of 5% heated tobacco product prevalence from the recent pilot cross-sectional survey of 4,154 TNP consumers. The authors need to clarify the ‘full sample’ that can calculate the 16% and 11%.
- The authors need to add the time frame (e.g. 2016-2017) of ‘results of a population-based survey on TNP’ in the first sentence of the last paragraph.
- The sentences following ‘From a tobacco harm reduction perspective’ explained the need to conduct the other cross-sectional survey based on IQOS purchasers. The reviewer suggests the author to add a sentence before ‘From a tobacco harm reduction perspective’ to introduce second cross-sectional survey instead of mentioning it at the end of the paragraph with ‘For these reasons’.

**Methods** 

*Study Design and Setting*

- The authors stated, ‘The current paper analyzed the data from the first year of the surveys, which comprised four waves of fieldwork.’ The author needs to clarify if this sentence and the following sentence are for both cross-sectional surveys. For example, the reviewer would like to know if the two cross-sectional surveys are conducted at the same time period (i.e. ‘in December 2016 and March, May, and July 2017’).

*Participants*

- In the IQOS User Survey section, the authors should mention it was the ‘Members of the PMI Japan IQOS Owner database *who agreed to be contacted for research purposes*’ that were ‘selected randomly and contacted by email.’

*Study Size*

- The reviewer suggests changing the sentence with ‘This number sufficed...’ to ‘This number sufficed to estimate a 95% confidence interval (CI) with a margin of error of ±0.275 percent given a population prevalence of IQOS use considered to be around 1%’. This suggestion also applies to the sentence in the IQOS User Survey.

*Analytical Methods*

- Clarifications are needed for the ‘average daily consumption of TNP’. There are two typical ways to report on average daily consumption: average usage per day *on days used* or usage per day *on 30-day average* (which is usage per day on days used*number of days used/30).

**Results**

*Sample Characteristics*

- In Table 1, the authors reported 27.5% don’t know/not applicable for the occupation distribution in the IQOS user survey. This ‘Don’t know/Not applicable’ level was not included in the Japanese population column nor the ‘General population survey’ column. The reviewer suggests either to exclude this level for IQOS user survey or include this level for the general population survey to make occupation distributions comparable.

*Prevalence of TNP Use in the general population survey*

- Given the TNP usage information is available, the reviewer suggests the authors to also report on exclusive, dual and poly use patterns among current IQOS users in the general population survey and compare the results with the use pattern from the IQOS user survey.
- The word ‘at least’ should be removed from the sentence that ‘Cigarette smoking was most prevalent in participants who had completed at least high school-level education….’

*Distribution of use patterns among TNP users in the IQOS user survey*

- The reviewer suggests adding the proportion of daily versus non-daily users, and average number of days used IQOS or cigarettes from the IQOS user survey. These would provide additional context when evaluating the use consumption of IQOS or cigarettes for dual and poly users. The reviewer believes the mean consumption per day is average use consumption on days used so the number of days used cigarette and IQOS would be extremely helpful to understand the dual and poly use behavior.

**Discussions**

- The reviewer suggests changing ‘could change as the majority of smokers in the population adopt the product’ to ‘could change as more smokers in the population adopt the product’.
- The reviewer suggests changing ‘low initiation and re-initiation of TNPs by non-users or former users, respectively’ to ‘low initiation and re-initiation of TNPs by *never users* or former users, respectively’ to align with language used in later paragraphs regarding never and former users.
- The reviewer suggests revising end of the sentence ‘…as more individuals adopt the use of IQOS the longer it is on the market.’

Is the work clearly and accurately presented and does it cite the current literature?

Yes

If applicable, is the statistical analysis and its interpretation appropriate?

Yes

Are all the source data underlying the results available to ensure full reproducibility?

Yes

Is the study design appropriate and is the work technically sound?

Yes

Are the conclusions drawn adequately supported by the results?

Yes

Are sufficient details of methods and analysis provided to allow replication by others?

Yes

Reviewer Expertise:

Statistics, Epidemiology, Population Modeling and Tobacco Survey Research.

I confirm that I have read this submission and believe that I have an appropriate level of expertise to confirm that it is of an acceptable scientific standard, however I have significant reservations, as outlined above.

# Response

**Published:** 2022-03-16

**Authors:** Esther Afolalu

**Affiliations:** PMI R&D, Switzerland

The authors presented the prevalence and patterns of tobacco and/or nicotine-containing products (TNP) usage based on first annual (2016-2017) data of two cross-sectional surveys in Japan. The survey methodology and sampling methods were clearly described. The analysis results provided insights of the early adoption and market expansion of IQOS in Japan. The reviewer has listed the comments for each section below. The reviewer has the suggestions to add analysis results regarding exclusive, dual and poly use patterns from both surveys (i.e. general population survey and IQOS user survey) and the cigarette and IQOS use patterns (e.g. daily use, number of days used) for exclusive and dual/poly users.

 Abstract

- The authors described that the analysis is based on ‘general adult population’ (and also mentioned ‘legal age’ in the Introduction section) while the analysis results were presented for 20 years of age or older. The authors should specify the age range of the baseline population upfront in the abstract as well as in the introduction or study design section as the ‘adult age’ or ‘legal age’ may differ by countries. 

***Thanks for the observation, this is now clarified in the abstract.***

 Introduction

- The authors stated ‘In the full sample, 16% reported exclusive use of heated tobacco products and 11% reported dual use with cigarettes’, following the mention of 5% heated tobacco product prevalence from the recent pilot cross-sectional survey of 4,154 TNP consumers. The authors need to clarify the ‘full sample’ that can calculate the 16% and 11%

***Thank you, now clarified that this sample refers to the sample of current tobacco users in the survey.***

- The authors need to add the time frame (e.g. 2016-2017) of ‘results of a population-based survey on TNP’ in the first sentence of the last paragraph.

***The time frame 2016-2017 now added for clarification.***

- The sentences following ‘From a tobacco harm reduction perspective’ explained the need to conduct the other cross-sectional survey based on IQOS purchasers. The reviewer suggests the author to add a sentence before ‘From a tobacco harm reduction perspective’ to introduce second cross-sectional survey instead of mentioning it at the end of the paragraph with ‘For these reasons’.

***Thank you, this is now updated.***

 Methods 

*Study Design and Setting*

- The authors stated, ‘The current paper analyzed the data from the first year of the surveys, which comprised four waves of fieldwork.’ The author needs to clarify if this sentence and the following sentence are for both cross-sectional surveys. For example, the reviewer would like to know if the two cross-sectional surveys are conducted at the same time period (i.e. ‘in December 2016 and March, May, and July 2017’).

***Now clarified in ‘Study design and Setting’ section that the timeframe of the fieldwork applied to both the general population and IQOS user sample surveys.***

*Participants*

- In the IQOS User Survey section, the authors should mention it was the ‘Members of the PMI Japan IQOS Owner database *who agreed to be contacted for research purposes*’ that were ‘selected randomly and contacted by email.’

***Thank you for this observation, this is now added for clarification.***

*Study Size*

- The reviewer suggests changing the sentence with ‘This number sufficed...’ to ‘This number sufficed to estimate a 95% confidence interval (CI) with a margin of error of ±0.275 percent given a population prevalence of IQOS use considered to be around 1%’. This suggestion also applies to the sentence in the IQOS User Survey.

***Thank you for noting this omission, this is now updated.***

*Analytical Methods*

- Clarifications are needed for the ‘average daily consumption of TNP’. There are two typical ways to report on average daily consumption: average usage per day *on days used* or usage per day *on 30-day average* (which is usage per day on days used*number of days used/30).

***Now specified that Average daily consumption of TNP (mean consumption per day) refers to average number of units/times of product use per day on days used in the past 30 days.***

 Results

*Sample Characteristics*

- In Table 1, the authors reported 27.5% don’t know/not applicable for the occupation distribution in the IQOS user survey. This ‘Don’t know/Not applicable’ level was not included in the Japanese population column nor the ‘General population survey’ column. The reviewer suggests either to exclude this level for IQOS user survey or include this level for the general population survey to make occupation distributions comparable.

***Thank you, we agree this can be excluded from IQOS survey. We also added a footnote to Table 2 for further clarification.***

*Prevalence of TNP Use in the general population survey*

- Given the TNP usage information is available, the reviewer suggests the authors to also report on exclusive, dual and poly use patterns among current IQOS users in the general population survey and compare the results with the use pattern from the IQOS user survey.

***Thanks for this suggestion, we have now added an additional Table (Table 3) to show distributions of patterns of TNP use among current TNP users in the general population sample and added a note on this comparison in the discussion.***

- The word ‘at least’ should be removed from the sentence that ‘Cigarette smoking was most prevalent in participants who had completed at least high school-level education….’

***The word ‘at least’ now removed for clarity.***

*Distribution of use patterns among TNP users in the IQOS user survey*

- The reviewer suggests adding the proportion of daily versus non-daily users, and average number of days used IQOS or cigarettes from the IQOS user survey. These would provide additional context when evaluating the use consumption of IQOS or cigarettes for dual and poly users. The reviewer believes the mean consumption per day is average use consumption on days used so the number of days used cigarette and IQOS would be extremely helpful to understand the dual and poly use behavior.

***Thank you for the suggestion, we have now clarified that mean consumption per day refers to average number of units/times of product use per day on days used in the past 30 days. We have also added a column to show the average number of days used in past 30 days.***

 Discussions

- The reviewer suggests changing ‘could change as the majority of smokers in the population adopt the product’ to ‘could change as more smokers in the population adopt the product’.

***This text is now updated.***

- The reviewer suggests changing ‘low initiation and re-initiation of TNPs by non-users or former users, respectively’ to ‘low initiation and re-initiation of TNPs by *never users* or former users, respectively’ to align with language used in later paragraphs regarding never and former users.

***Thank you, this text is now updated.***

- The reviewer suggests revising end of the sentence ‘…as more individuals adopt the use of IQOS the longer it is on the market.’

***This sentence has now been rephrased.***
